# Supplementary material for: Genetic Dissection of Temperament Personality Traits in Italian Isolates
Source: Genes (Basel). 2021 Dec 21;13(1):4. doi: 10.3390/genes13010004 (PMC8774962; doi:10.3390/genes13010004)

Novelty Seeking, NS

NS

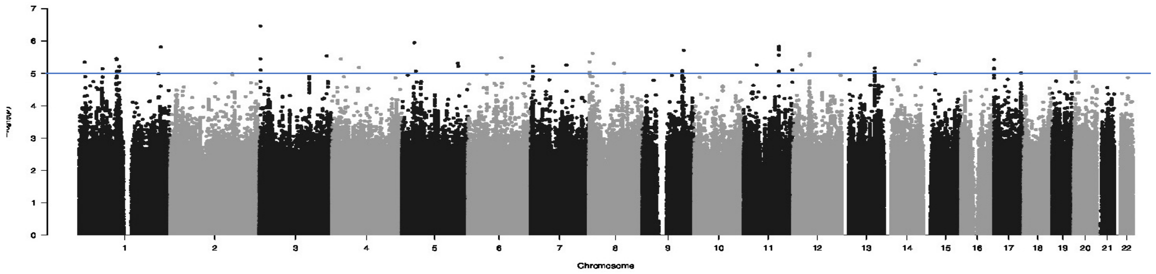

NS1

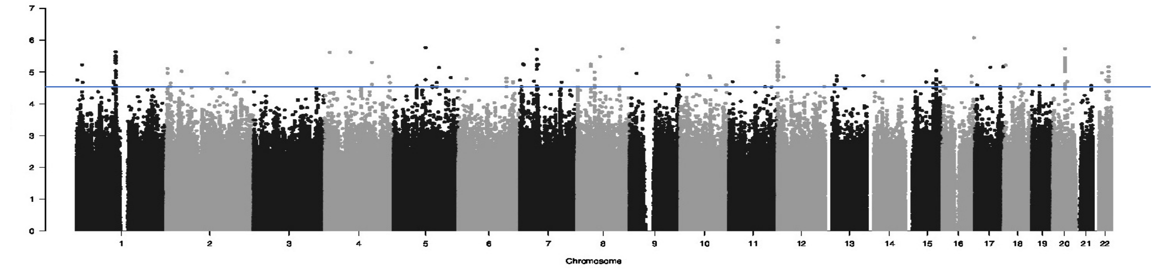

NS2

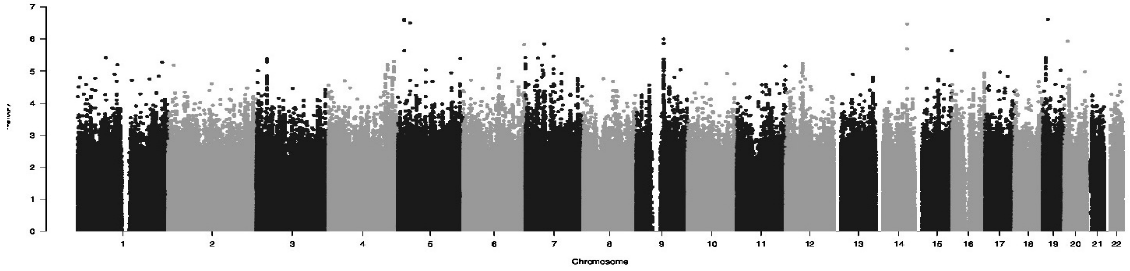

NS3

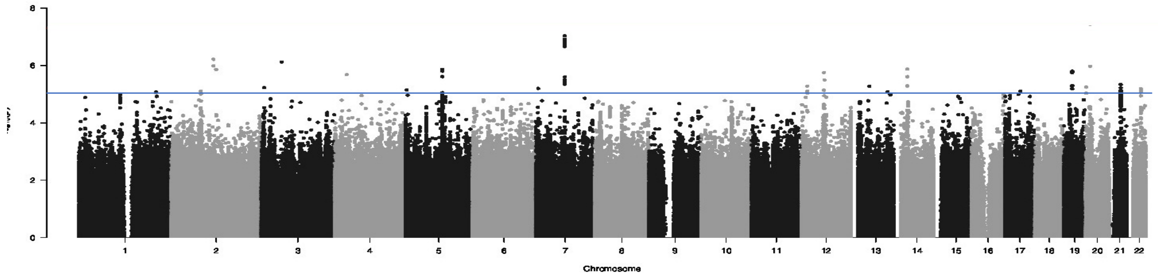

NS4

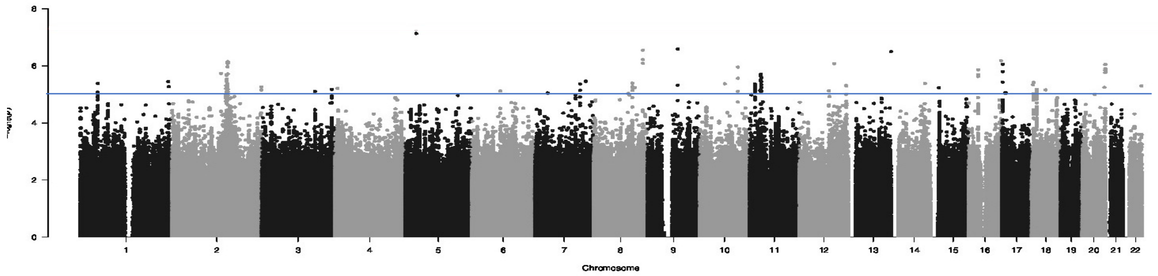

Novelty Seeking, NS

NS

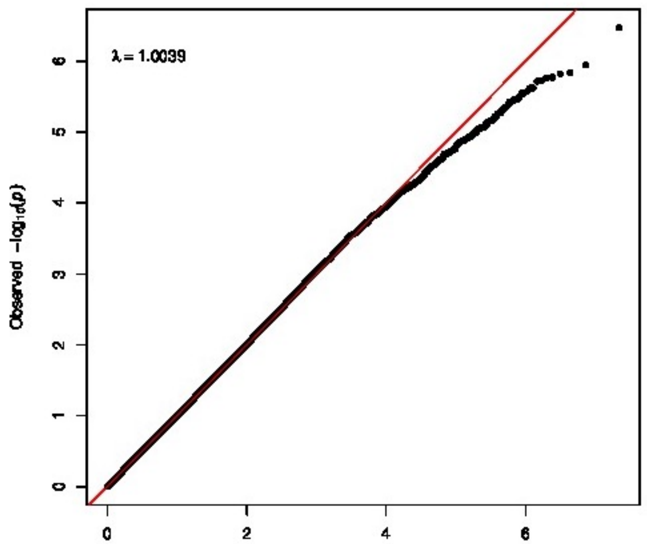

NS2

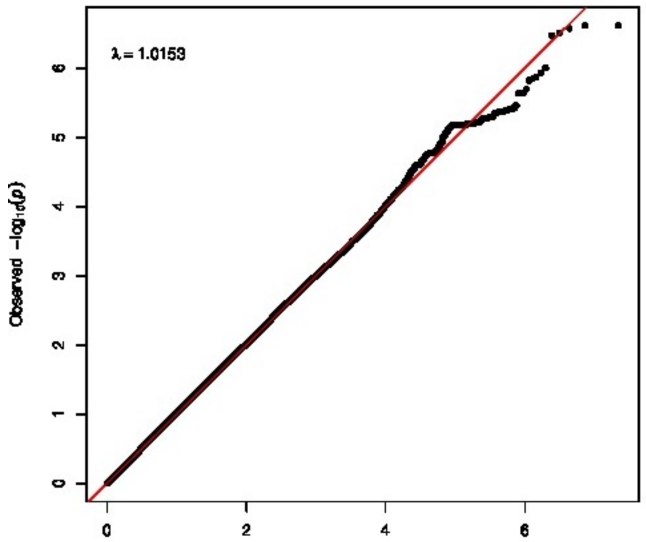

NS4

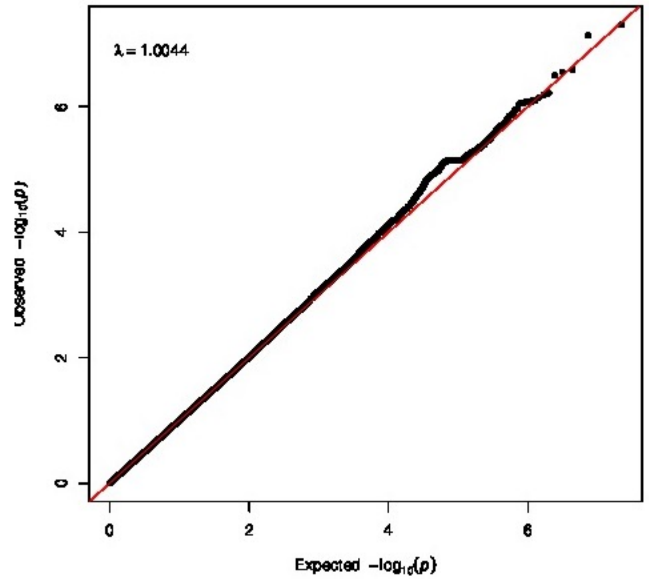

NS1

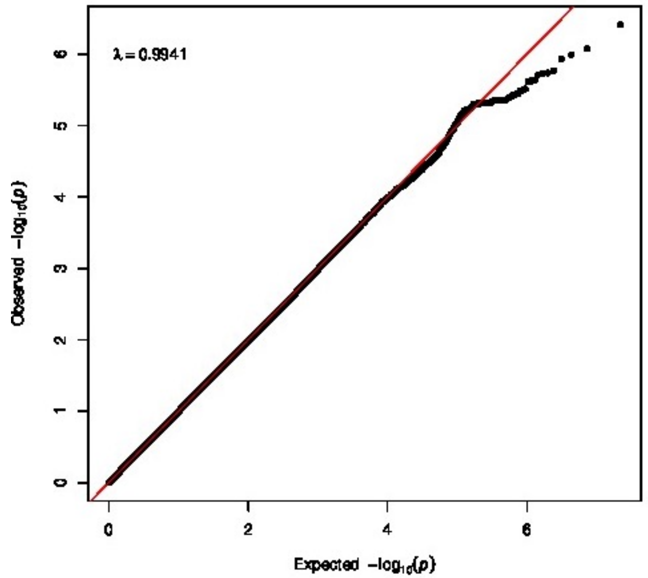

NS3

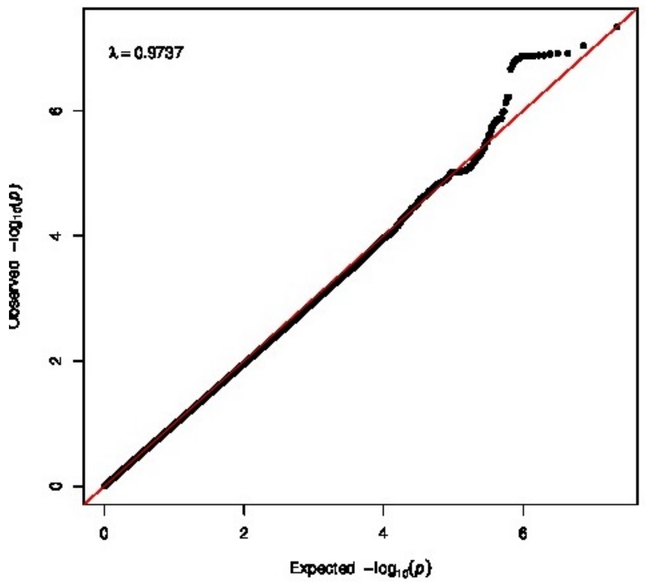

Harm Avoidence, HA

HA

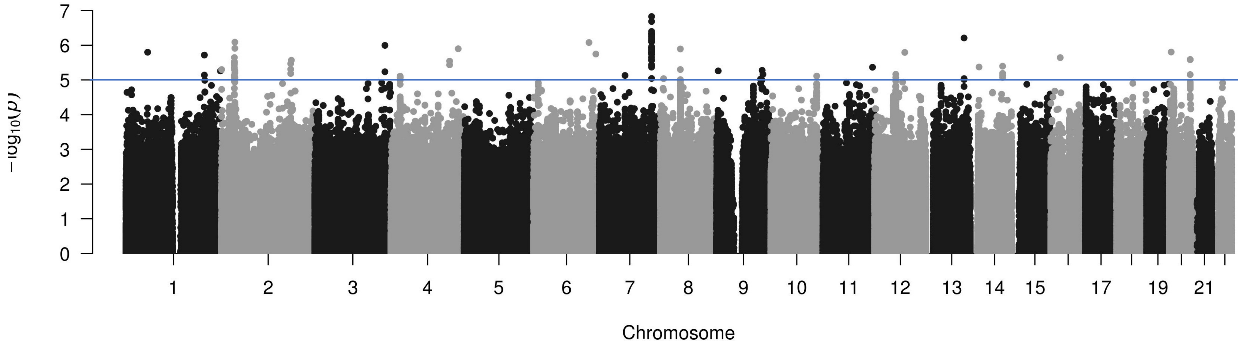

HA3

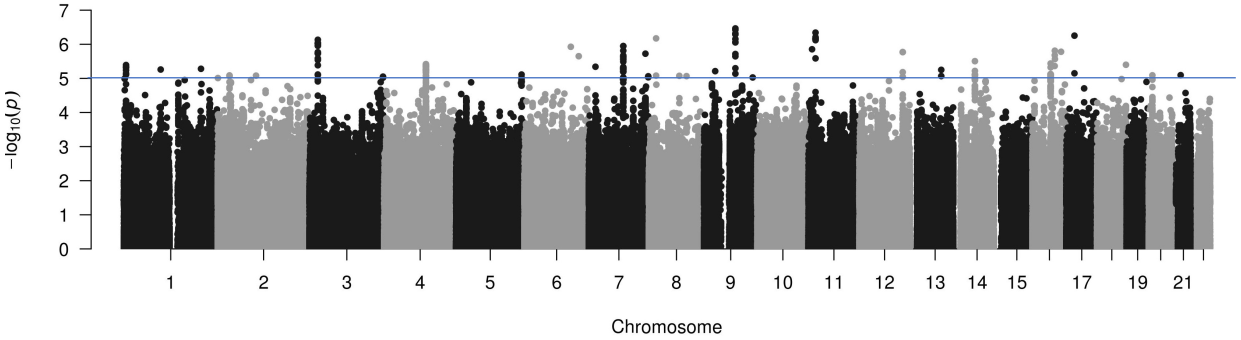

HA1

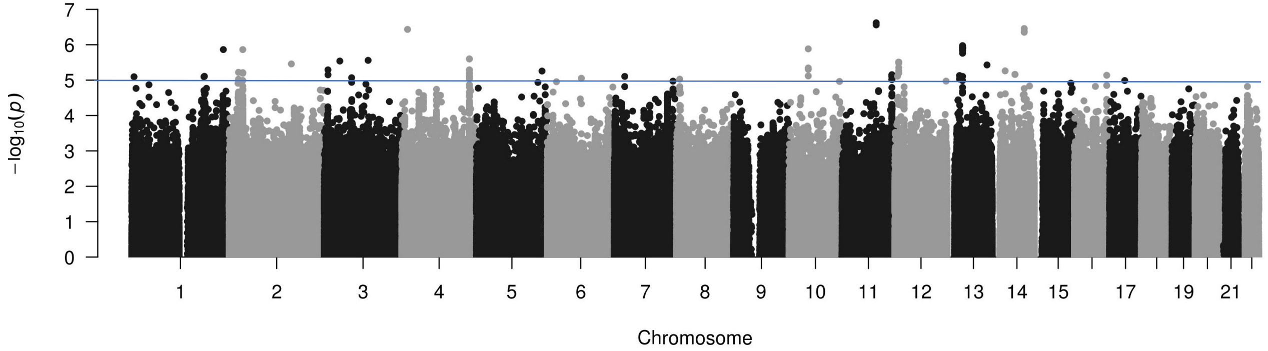

HA4

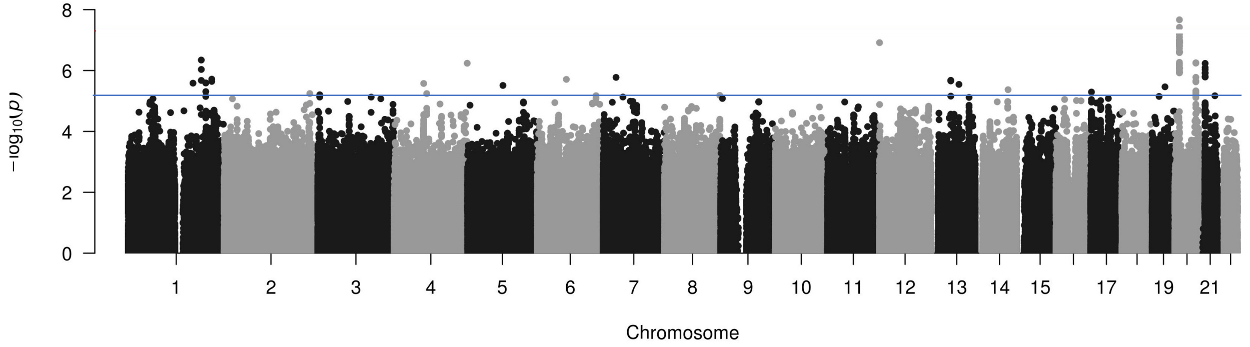

HA2

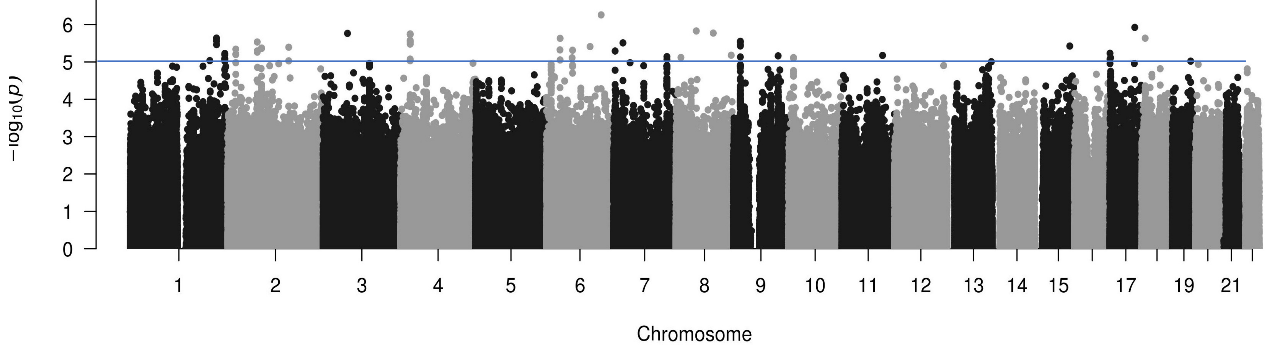

Harm Avoidence, HA

HA

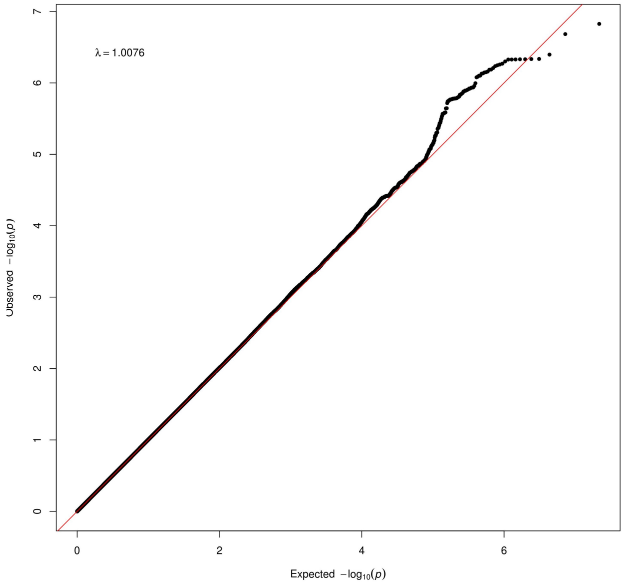

HA2

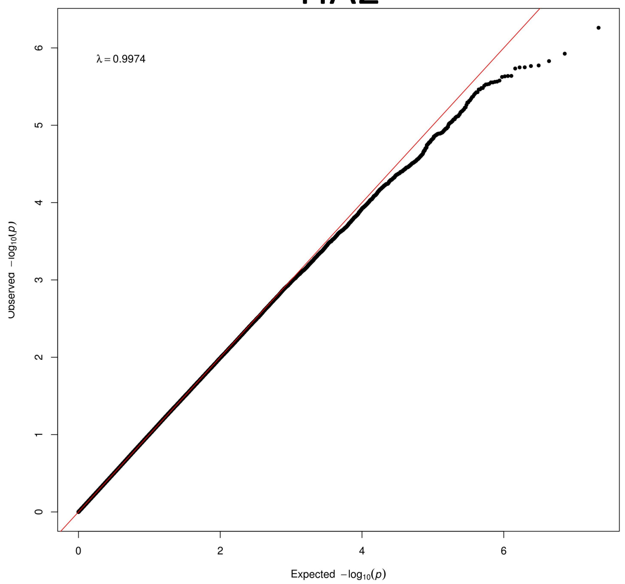

HA4

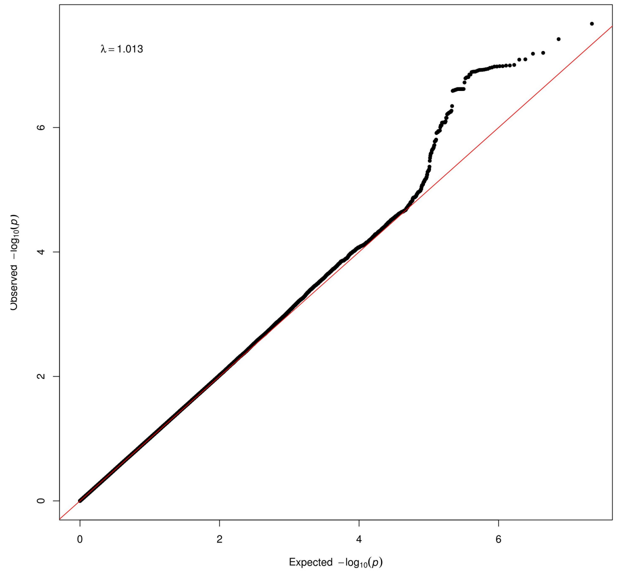

HA1

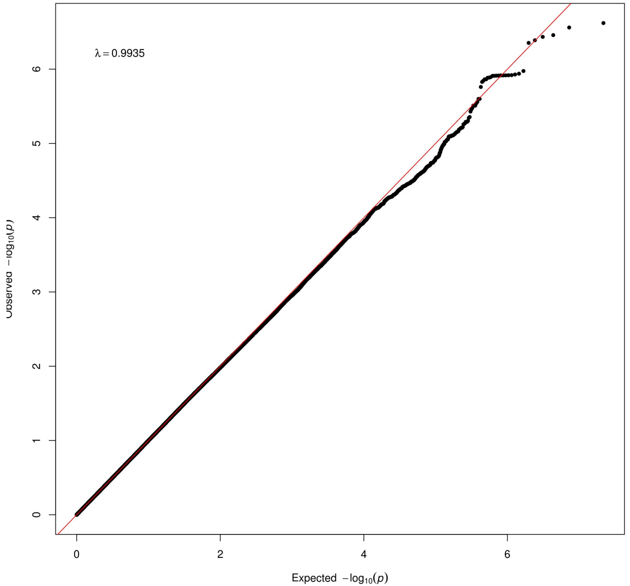

HA3

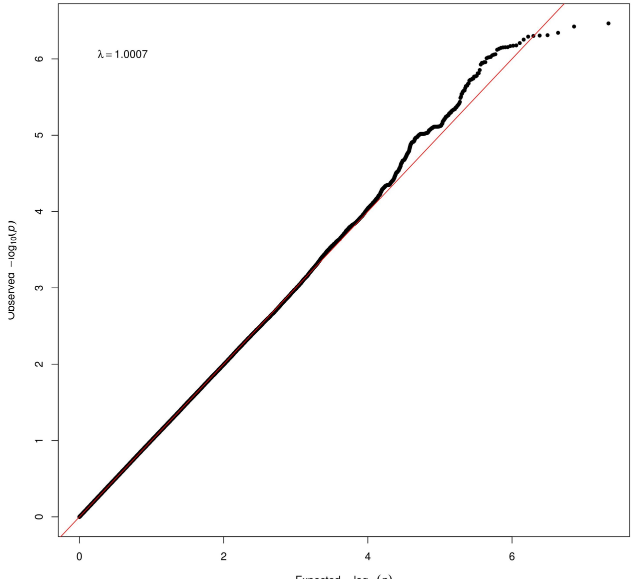

Reward Dependence, RD

RD

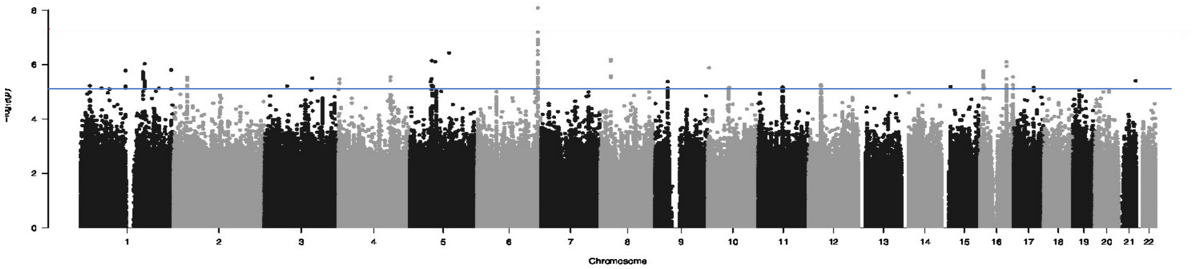

RD3

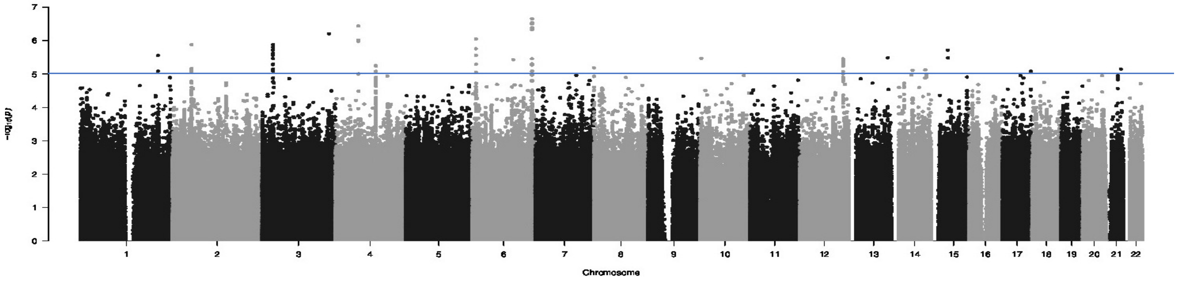

RD1

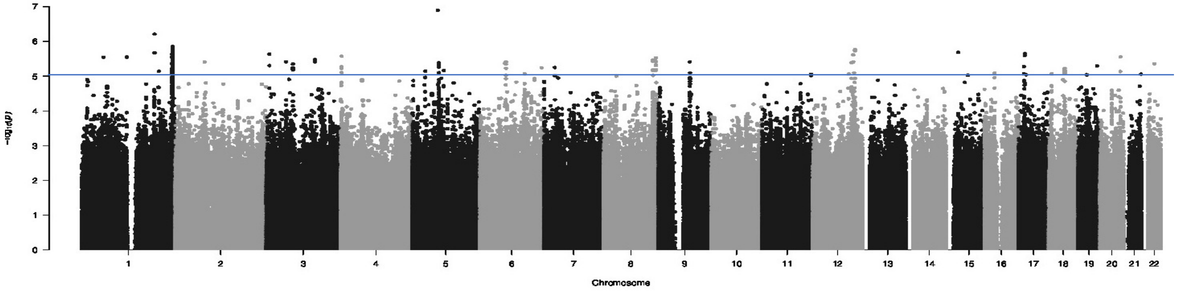

RD4

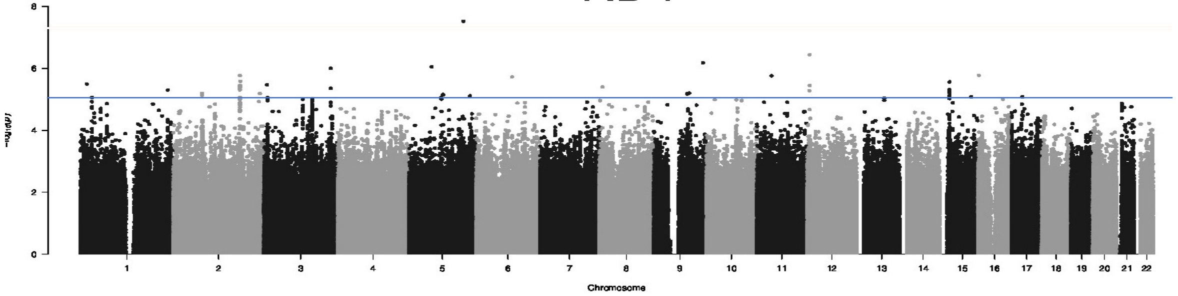

Reward Dependence, RD

RD

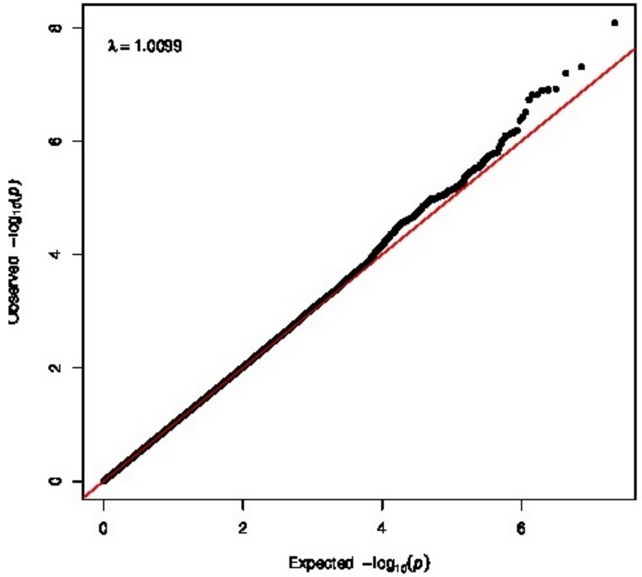

RD3

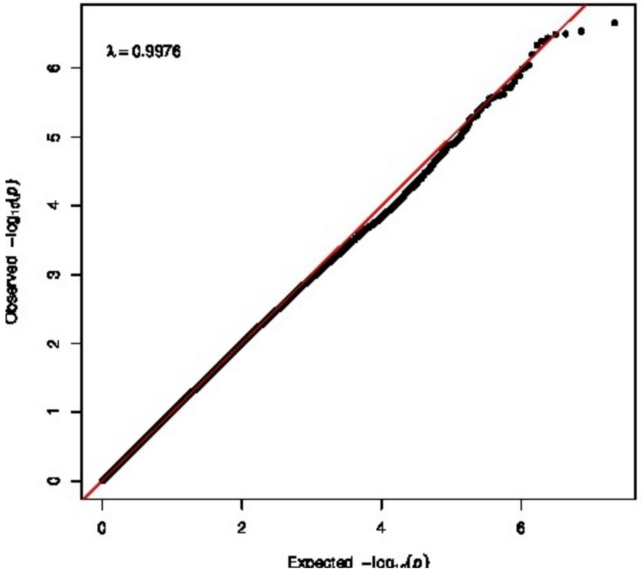

RD1

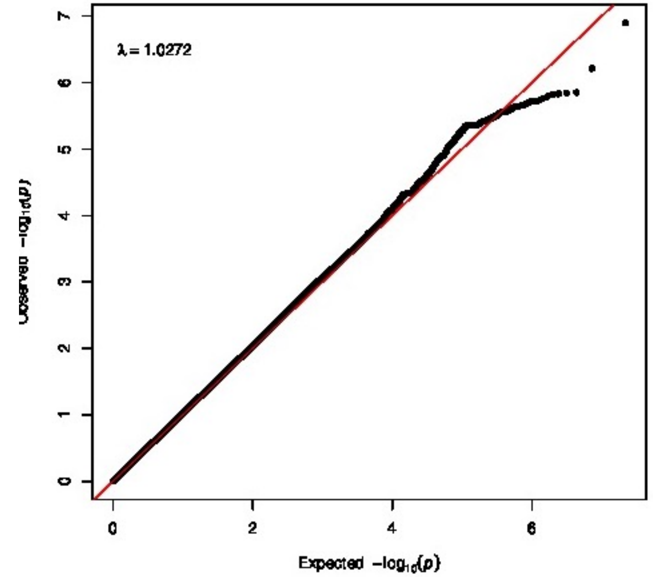

RD4

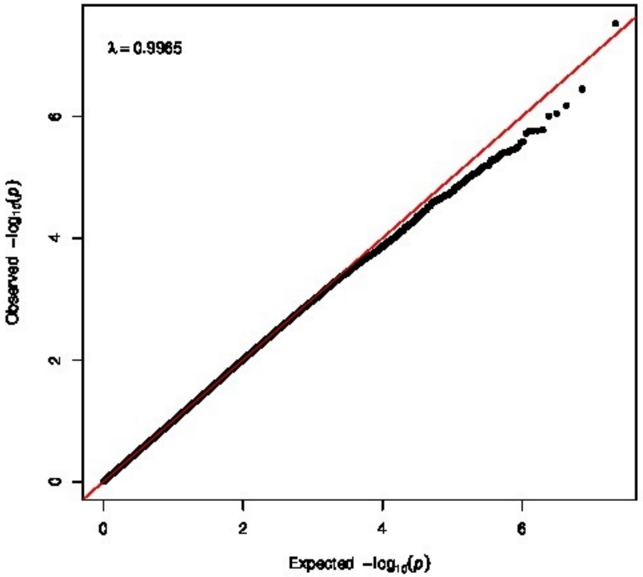

Persistence, P

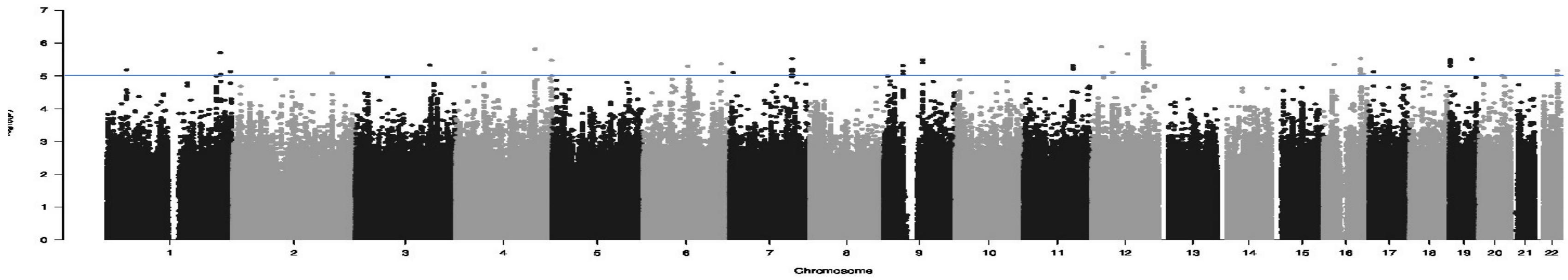

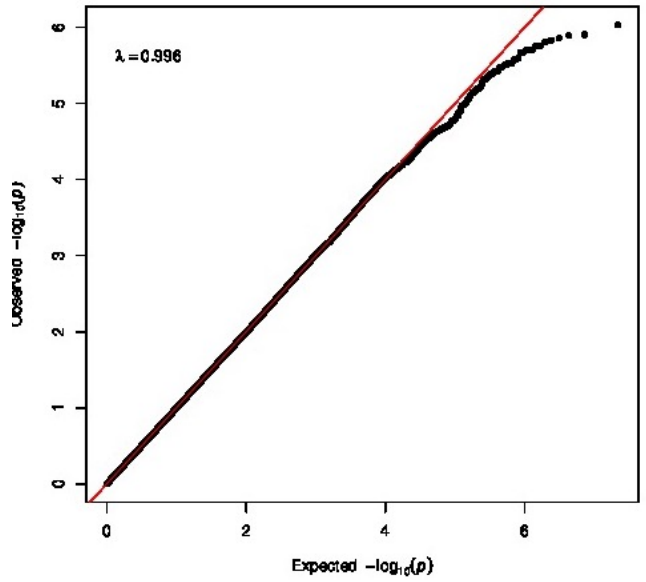

Supplement: Supplementary file 1 [file genes-13-00004-s001.zip › Figure S1.pdf]
